# Supplementary material for: The causal relationship between the human gut microbiota and pyogenic arthritis: a Mendelian randomization study
Source: Front Cell Infect Microbiol. 2024 Nov 26;14:1452480. doi: 10.3389/fcimb.2024.1452480 (PMC11629706; doi:10.3389/fcimb.2024.1452480)
Supplement: Supplementary file 1 [file DataSheet1.zip › mendelian test/ebi-a-GCST90027839.csv_leave_one_out.pdf]

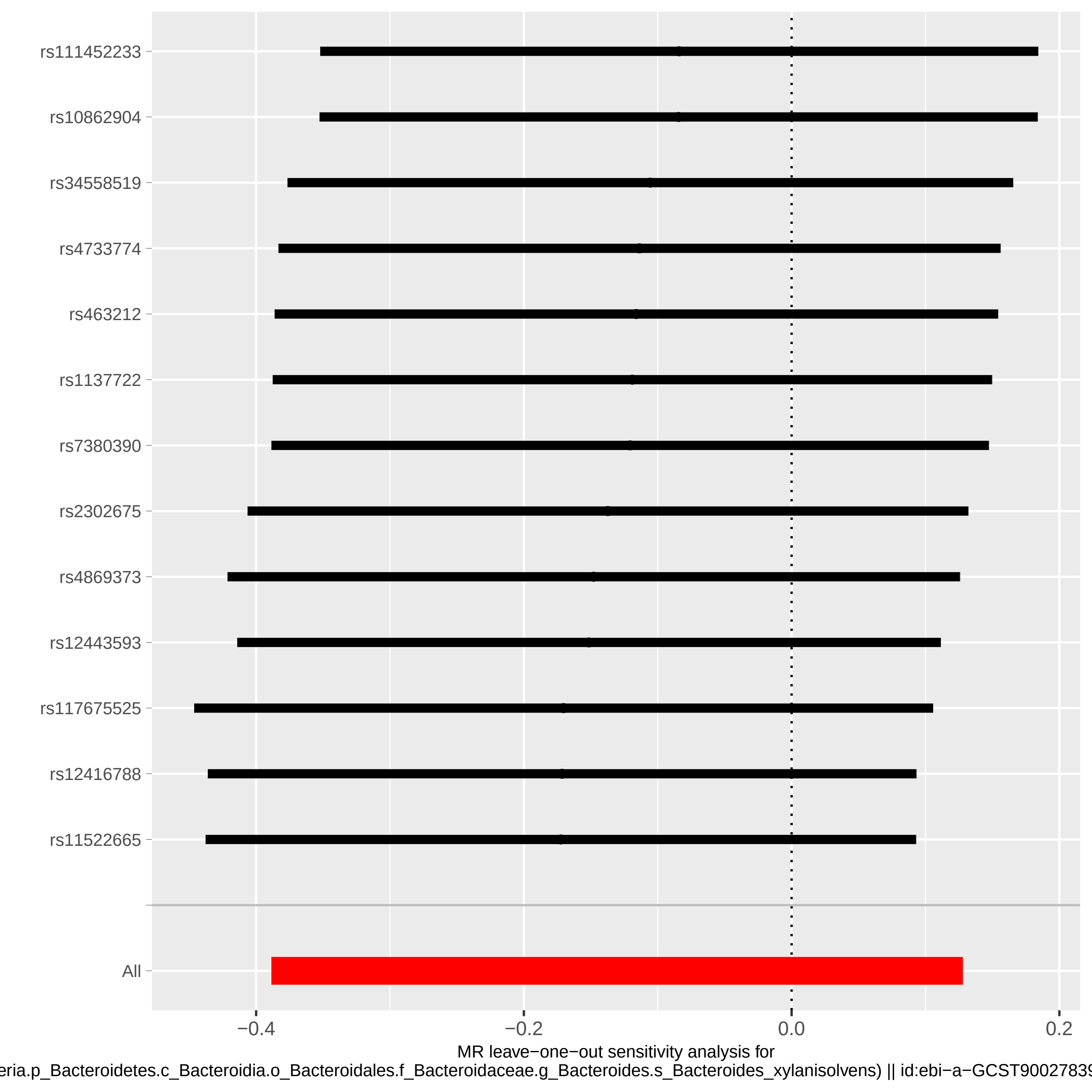

rs111452233
rs10862904
rs34558519
rs4733774
rs463212
rs1137722
rs7380390
rs2302675
rs4869373
rs12443593
rs117675525
rs12416788
rs11522665
All
−0.4
−0.2
0.0
0.2
MR leave−one−out sensitivity analysis for
eria.p_Bacteroidetes.c_Bacteroidia.o_Bacteroidales.f_Bacteroidaceae.g_Bacteroides.s_Bacteroides_xylanisolvens) || id:ebi−a−GCST9002783
